# Sevoflurane postconditioning attenuates cardiomyocytes hypoxia/reoxygenation injury via PI3K/AKT pathway mediated HIF-1α to regulate the mitochondrial dynamic balance

**DOI:** 10.1186/s12872-024-03868-1

**Published:** 2024-05-29

**Authors:** Haiping Ma, Tianliang Hou, Jianjiang Wu, Jiyao Zhao, Haoran Cao, Maisitanguli. Masula, Jiang Wang

**Affiliations:** The First Affiliated Hospital of Xinjiang Medical University, 393 Xinyi Road, Xinjiang Uygur Autonomous Region, Urumqi, 830000 China

## Abstract

**Background:**

Myocardial ischemia-reperfusion injury (I/RI) is a major cause of perioperative cardiac-related adverse events and death. Studies have shown that sevoflurane postconditioning (SpostC), which attenuates I/R injury and exerts cardioprotective effects, regulates mitochondrial dynamic balance via HIF-1α, but the exact mechanism is unknown. This study investigates whether the PI3K/AKT pathway in SpostC regulates mitochondrial dynamic balance by mediating HIF-1α, thereby exerting myocardial protective effects.

**Methods:**

The H9C2 cardiomyocytes were cultured to establish the hypoxia-reoxygenation (H/R) model and randomly divided into 4 groups: Control group, H/R group, sevoflurane postconditioning (H/R + SpostC) group and PI3K/AKT blocker (H/R + SpostC + LY) group. Cell survival rate was determined by CCK-8; Apoptosis rate was determined by flow cytometry; mitochondrial membrane potential was evaluated by Mito Tracker™ Red; mRNA expression levels of AKT, HIF-1α, Opa1and Drp1 were detected by quantitative real-time polymerase chain reaction (qRT-PCR); Western Blot assay was used to detect the protein expression levels of AKT, phosphorylated AKT (p-AKT), HIF-1α, Opa1 and Drp1.

**Results:**

Compared with the H/R group, the survival rate of cardiomyocytes in the H/R + SpostC group increased, the apoptosis rate decreased and the mitochondrial membrane potential increased. qRT-PCR showed that the mRNA expression of HIF-1α and Opa1 were higher in the H/R + SpostC group compared with the H/R group, whereas the transcription level of Drp1 was lower in the H/R + SpostC group. In the H/R + SpostC + LY group, the mRNA expression of HIF-1α was lower than the H/R + SpostC group. There was no difference in the expression of Opa1 mRNA between the H/R group and the H/R + SpostC + LY group. WB assay results showed that compared with the H/R group, the protein expression levels of HIF-1α, Opa1, P-AKT were increased and Drp1 protein expression levels were decreased in the H/R + SpostC group. HIF-1α, P-AKT protein expression levels were decreased in the H/R + SpostC + LY group compared to the H/R + SpostC group.

**Conclusion:**

SpostC mediates HIF-1α-regulated mitochondrial fission and fusion-related protein expression to maintain mitochondrial dynamic balance by activating the PI3K/AKT pathway and increasing AKT phosphorylation, thereby attenuating myocardial I/R injury.

**Supplementary Information:**

The online version contains supplementary material available at 10.1186/s12872-024-03868-1.

## Introduction

Myocardial ischemia-reperfusion injury (I/RI) is a common clinical pathophysiological process that is the main reason for the occurrence and death of the perioperative adverse cardiac events and death [1]. Sevoflurane postconditioning (SPostC) has been confirmed to exert good myocardial protective effects against myocardial I/RI by inhibiting apoptosis, reducing myocardial LDH content, decreasing ROS production, and increasing ATP content, and is a potential treatment against myocardial injury in the perioperative period [2–4].

Cardiomyocytes are high energy consuming organs, of which mitochondria account for approximately 30%. Mitochondria provide 95% of the adenosine triphosphate (ATP) generated by oxidative phosphorylation (OXPHOS) for the beating heart and act as the metabolic center for the citric acid cycle and fatty acid β-oxidation [5–7]. Mitochondria are highly dynamic organelles that constantly undergo fusion and fission to achieve changes in their shape, size, number, position and function [8]. It has been shown that the balance of mitochondrial fission and fusion is disrupted after myocardial exposure to I/RI, leading to increased myocardial damage [9]. Hypoxia-inducible factors (HIF-1α), as initiators of endogenous protective mechanisms that regulate the initiation of myocardial hypoxia, are considered to be the main regulators of anti-hypoxic damage [10]. Our previous study confirmed that SPostC exerts myocardial protective effects by regulating mitochondrial fission and fusion through HIF-1α, maintaining mitochondrial homeostasis and reducing I/RI [11], but the exact mechanism is unclear.

The PI3K/AKT signal pathway, an important component of the RISK signal pathway, is involved in the regulation of a variety of cellular activities, including cell survival, proliferation, metabolism, neuroscience, motility and cancer progression [12, 13]. It has been demonstrated that in the rat heart, SPostC exerts a cardioprotective effect against I/RI through activation of the PI3K/AKT signal pathway [14]. Numerous studies have shown that PI3K/Akt/HIF-1α pathway is frequently activated in human cancers and plays a key role in promoting glycolysis [15–18]. However, it is unclear whether the PI3K/AKT signal pathway is involved in the regulation of mitochondrial fission and fusion by SPostC via HIF-1α.Thus, we investigated whether SpostC regulates the balance of mitochondrial dynamics and reduces myocardial I/RI via the PI3K/AKT/HIF-1α pathway.

## Materials & methods

### Cell culture and processing

The H9C2 rat embryonic cardiomyocyte line was obtained from keyGEN BioTECH. Cell culture conditions were DMEM (low glucose, 5µM) medium + 10% (v/v) FBS (Gibco, USA) + 1% (v/v) Penicillin/Streptomycin solution (Gibco, USA), 37 °C, 5% CO_2_, saturation humidity. H9C2 cells were passaged at a ratio of 1:4 in 55 cm^2^ culture plate when the cell fusion reached 90% (estimated by visual observation). Cells were incubated in 5% CO_2_ incubator at 37 ℃ for 48 h. When the cells grew to 90% fusion, the supernatant was discarded, then the cells were gently washed twice with PBS, and serum-free DMEM (low sugar, 5 mM) medium was added. The plates were placed in a saturated tri-gas incubator containing 95%N_2_ and 5%CO_2_ for 3 h at 37 ℃. After incubation under hypoxic conditions, the supernatant was discarded and replaced with fresh serum-free DMEM (low glucose, 5µM) medium containing 10% FBS. The cells were then reoxygenated was done by incubation in 95% air and 5% CO_2_ at 37 ℃ for more than 3 h.

### Sevoflurane postconditioning (SpostC) procedure

According to the previous studies (Yang et al., 2016, Ma et al., 2020), the sevoflurane post-treatment was applied for 15 min before the start of reoxygenation. using a sevoflurane volatile canister, the outlet of the volatile canister was connected closely to the inlet of the portable incubator, the flow rate was adjusted to 2 L/min and the concentration of sevoflurane was 2.4% for 10 min, then the inlet and outlet were closed. The cells to be treated were incubated in a triple gas incubator filled with sevoflurane at 37 °C for 15 min and then removed and continued to be incubated in CO_2_ incubator for 165 min.

### Investigation of drug intervention concentrations

**Fig. 1:**
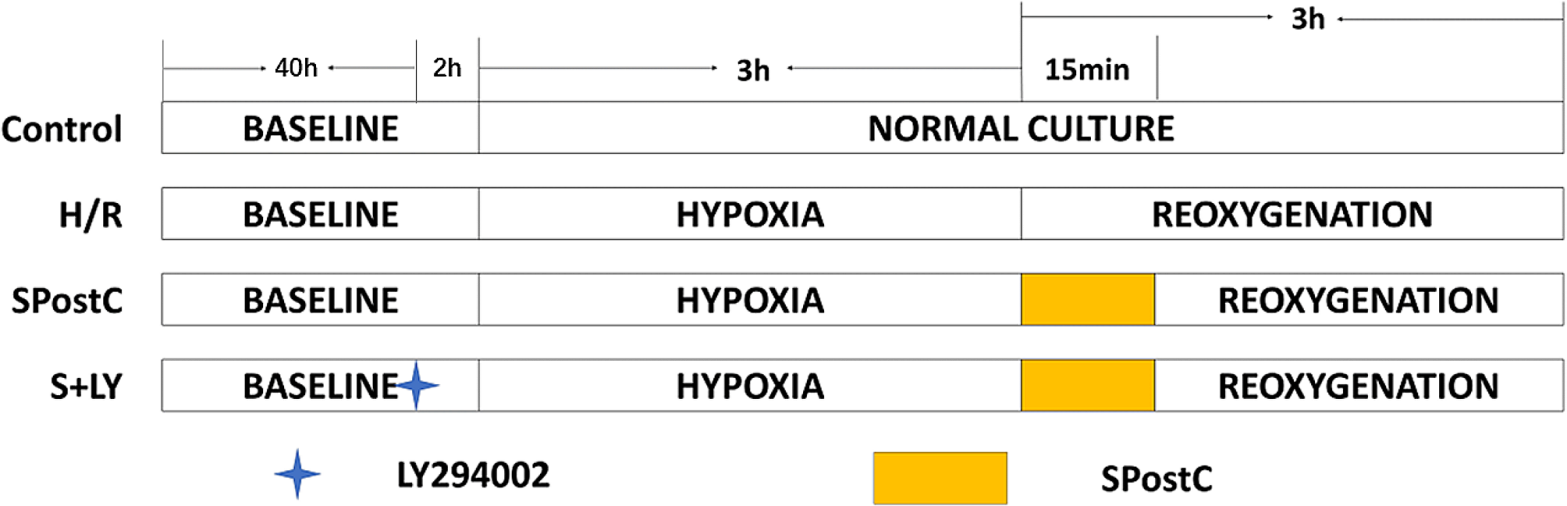
Effect of H/R injury, SpostC and PI3K/AKT inhibitors on H9C2 cells. (**A**)Cell viability. (**B**) Cell survival rate. (**C**, **D**) Flow cytometry to measure apoptosis *: Compared with Control group, *P* < 0.05; **: Compared with Control group, *P* < 0.01; ***: Compared with Control group, *P* < 0.001; &: Compared with H/R group, *P* < 0.05; &&: Compared with H/R group, *P* < 0.01; &&&: Compared with H/R group, *P* < 0.001; ^^^: Compared with H/R + SpostC group, *P* < 0.001

Well-grown H9C2 cells with 90% fusion rate were digested with trypsin to prepare a single cell suspension of 5 × 10^4^ cells/ml in complete medium, then inoculated in 96-well plates (100µL/well, 5 × 10^3^ cells/well) and incubated at 37 °C with 5% CO_2_ for 24 h. Different concentrations of LY294002 (2.5, 5, 10, 20, 40 and 80 µM) were used for the intervention, while a control group was set up. After 8 h of intervention, the medium was discarded and 100 µL of the configured 10% CCK-8 solution was added to each well, and the incubation was continued in the incubator. 1 h later, the OD value at 450 nm was measured with an enzyme marker to detect the cell survival rate. It was found that 10 μm LY294002 had no significant effect on cell growth and was the optimal concentration for intervention (Fig. 1A).

### Experimental protocol

**Fig. 2:**
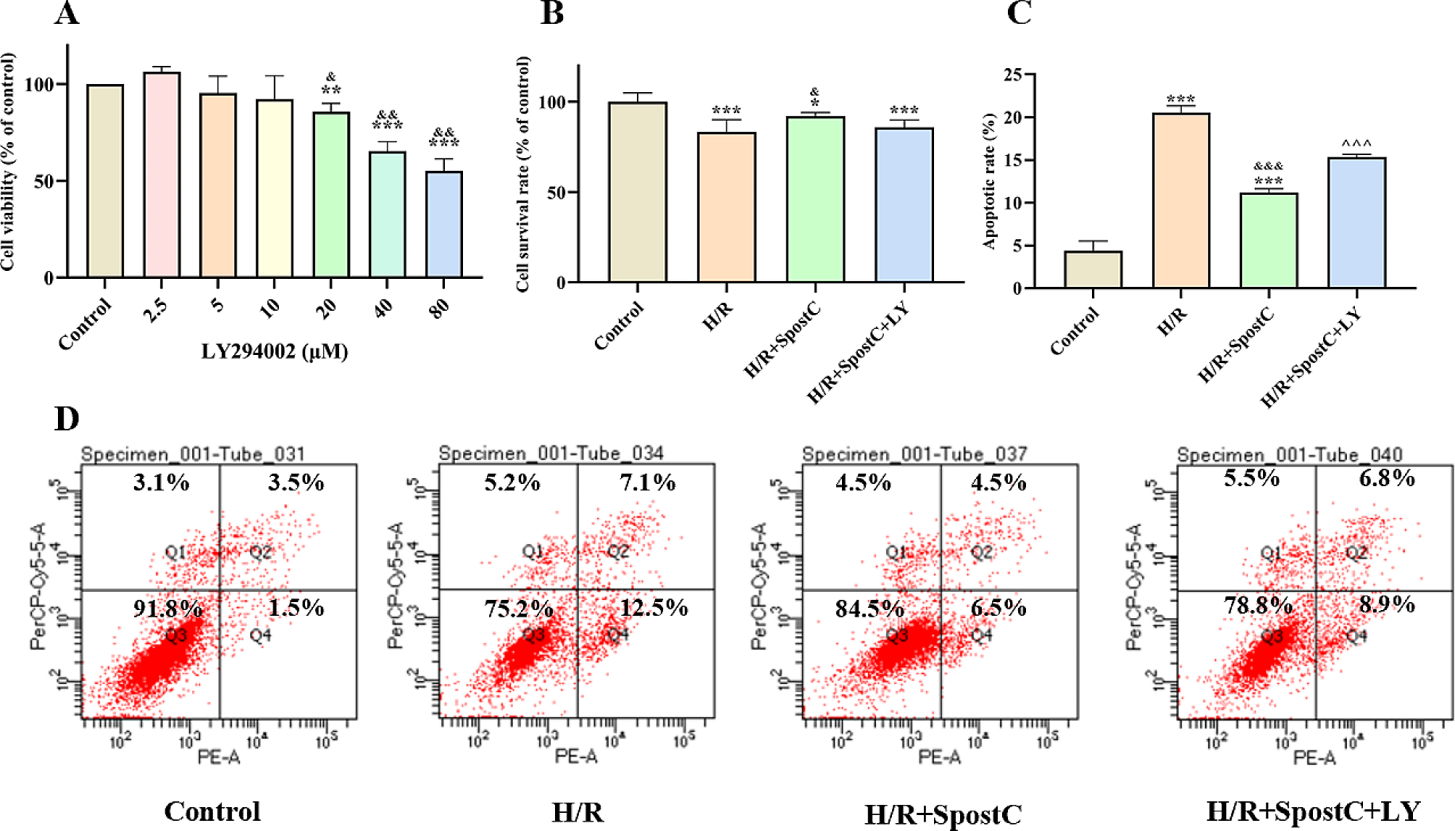
Grouping and flow chart

H9C2 cardiomyocytes were randomly divided into four groups (Fig. 2).

1. Control (Control) group: H9C2 cells incubated normally in the CO_2_ incubator for 48 h without any interventions.
2. Hypoxia/reoxygenation (H/R) group: H9C2 cells were incubated normally in CO_2_ incubator for 42 h and then placed in a tri-gas incubator for 3 h hypoxia and 3 h reoxygenation.
3. Sevoflurane postconditioning (H/R + SpostC) group: After 42 h of normal incubation in the CO_2_ incubator, H9C2 cells were placed in a tri-gas incubator for 3 h of hypoxia. Reoxygenation was started before SpostC for 15 min, followed by a further 165 min of incubation in the CO_2_ incubator.
4. PI3K/AKT inhibitor (H/R + SpostC + LY) group: After 40 h of normal incubation in the CO_2_ incubator, H9C2 cells were cultured in medium containing 10µM LY294002 until the end of the experiment, for 8 h. After 2 h of intervention, the cells were placed in a triple gas incubator for 3 h of hypoxia and SPostC for 15 min before the start of reoxygenation, followed by continued incubation in a CO_2_ incubator for 165 min.

### Determination of cell viability

A single cell suspension of 5 × 10^4^ cells/mL was prepared by digesting well-grown, 90% fused H9C2 cells with trypsin and cultured using complete medium. The suspension was inoculated into 96-well plates (100µL/well, 5 × 10^3^Cells/well). Cells were incubated at 37 °C and 5% CO_2_ for 24 h and then grouped according to experiment. Discarded the medium, added 100µL of 10% CCK-8 solution to each well and continued to incubate in the incubator. After 1 h, measured the OD value at 450 nm with a microplate reader to detect the cell survival rate.

### Flow cytometry assay

The percentage of cells apoptosis was determined by PE annexin-V apoptosis detection kit I. (BD Biosciences, USA) according to the manufacturer’s instructions as previously described (Ma et al., 2020).H9C2 cells in good growth condition with 90% confluence were digested with trypsin and prepared into a single cell suspension of 5 × 10^4^ cells/mL in complete medium and inoculated into 6-well plates; after treatment in experimental groups, the culture fluid from each group of cell bottles was aspirated together with the cells into a centrifuge tube (containing apoptotic or necrotic cells already in suspension) and centrifuged at 1000 rpm for 5 min. The supernatant was discarded. Wash twice with pre-cooled PBS and discard the supernatant. Add 500µL of 1×Binding Buffer to resuspend the cells and pass through a 200 Mesh sieve to make a single cell suspension. Add 5µL of Annexin V-PE and 10µL of 7-AAD to each tube, mix gently and leave for 5 min at 4°C, protected from light. Flow cytometry was performed within 30 min.

### Detection of mitochondrial membrane potential

We assessed mitochondrial membrane potential (Δψm) using Mito Tracker™ Red (Thermo Fisher Scientific, USA).H9C2 cells in good growth condition with 90% confluence were trypsinized and prepared into a single cell suspension of 5 × 10^4^ cells/mL with complete medium and inoculated onto adherent slides; cells were washed twice with PBS and labeled with 50nM MitoTracker™ Red (red), incubated for 20 min in the incubator. The cells were washed twice with PBS, the nuclei were labelled with 1 µg/ml Hoechst 33,342 working solution (blue), the cells were washed twice with PBS, the slides were fixed and sealed, and the mitochondria were observed by laser confocal microscopy and photographed.

### qRT-PCR detection

Total RNA was extracted using the Trizol reagent (Thermo Fisher Scientific, USA). Reverse transcription was performed using a 5X All-In-One RT MasterMix (Applied Biological Materials, CAN) and RT-PCR was performed using EvaGreen Express 2×qPCR MasterMix-Low Rox (Applied Biological Materials, CAN). The ΔΔC_t_ method was used for the calculation of relative changes in gene expression. The primer sequences for RT-PCR were listed in Table 1.

### Western blot analysis

Total protein was extracted using RIPA buffer with protease inhibitors (Boster, USA). For immunoblot analysis, protein samples were resolved by SDS-PAGE and transferred onto PVDF membranes by Mini-PROTEAN Tetra system (Bio-Rad Laboratories, USA). PVDF membranes were blocked in 1×TBST containing 5% non-fat dry milk for 1 h and incubated with primary antibodies overnight at 4 °C. PVDF membranes were washed 3 times with TBST and incubated with HRP-conjugated secondary antibodies (Abcam, UK) for 1 h at room temperature. SuperSignal™ West Pico PLUS Chemiluminescent Substrate (Thermo Fisher Scientific, USA) was used for visualization and imaging. Signals were detected and quantified using Image Lab 4.0 software (Bio-Rad Laboratories, USA). The primary antibodies were listed in Table 2.

**Table 1:** Primer sequences used in real time quantitative RT-PCR

Name of primers	The sequence of primers (5’ to 3’)	Product length
HIF-1α forward	GCAACTGCCACCACTGATGA	152 bp
HIF-1α reverse	GCTGTCCGACTGTGAGTACC	
OPA1 forward	CTGGGATCTGCTGTTGGAGG	251 bp
OPA1 reverse	GGTGTACCCGCAGTGAAGAA	
Drp1 forward	TGGAAAGAGCTCAGTGCTGG	149 bp
Drp1 reverse	CAACTCCATTTTCTTCTCCTGTTGT	
AKT1 forward	ATGAGAAGAAGCTGAGCCCAC	133 bp
AKT1 reverse	ACACACTCCATGCTGTCATCT	
Actin forward	CCCATCTATGAGGGTTACGC	150 bp
Actin reverse	TTTAATGTCACGCACGATTTC	

**Table 2:** Antibodies used in western blot

Primary antibody	Vendor	Catalog#	Concentration
β-actin	Sino Biological	100,166-MM10	1/15,000
HIF-1α	Abcam	Ab1	1/5000
Opa1	Abcam	ab157457	1/5000
Drp1	Abcam	ab184247	1/5000
AKT	CST	4691 S	1/5000
P-AKT	CST	9271 S	1/5000

### Statistical analysis

Statistical analysis was performed using SPSS19.0. Data are expressed as mean ± SD. One-way analysis of variance (ANOVA) was used for statistical analysis of data within and between groups, followed by the least significant difference (LSD) test for multiple comparisons of group means. *P* < 0.05 was considered statistically significant.

## Results

### The cardioprotective effect of SpostC was attenuated by PI3K/AKT blockader

In this study, CCK-8 was used to detect the survival rate of cardiomyocytes, and the results of the survival rate of cells in each group showed (Fig. 1B) that the cell survival rate was significantly decreased in the other three groups group compared with the control group. Cell survival was significantly increased in the H/R + SpostC group compared with the H/R group, and this protection was attenuated by the PI3K/AKT blocker (LY294002) in the H/R + SpostC group.

By flow cytometry, the other three groups showed an increase in apoptosis compared to the Control group; whereas the SpostC and SpostC + LY groups showed the opposite trends compared with the H/R group (Fig. 1C, D). The results showed that SPostC significantly increased myocardial survival and reduced myocardial apoptosis against attenuated myocardial H/RI after H/R injury in cardiomyocytes, whereas PI3K/AKT blockers diminished the cardioprotective effect.

### SPostC restores the loss of mitochondrial membrane potential caused by H/R damage

**Fig. 3:**
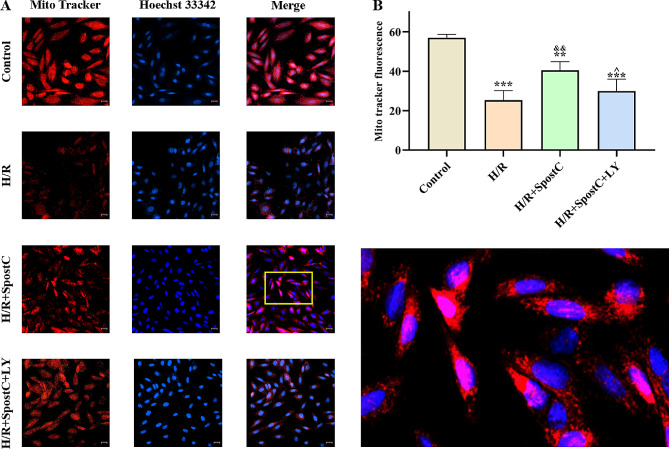
Fluorescence intensity histogram of mitochondria. (**A**) Representative images of Mito Tracker™ Red were capture using a laser scanning confocal microscope in H9C2 cells (200×). (**B**)The Histogram of mitochondrial fluorescence intensity in H9C2 cells. **: Compared with Control group, *P* < 0.01; ***: Compared with Control group, *P* < 0.001; &&: Compared with H/R group, *P* < 0.01; ^: Compared with H/R + SpostC group, *P* < 0.05

MitoTracker™ Red CMXRos is a red fluorescent dye used to stain mitochondria of living cells, and the accumulation of this dye depends on the membrane potential. Figure 3A shows a typical MitoTracker™ Red fluorescent image, the results showed that the fluorescence intensity was significantly decreased in the H/R group compared to the control group; the fluorescence intensity was significantly increased in the H/R + SPostC group compared to the H/R group; however, the fluorescence intensity was significantly decreased in the H/R + SpostC + LY group compared to the H/R + SPostC group (Fig. 3B). It is suggested that SpostC alters the mitochondrial membrane potential of cardiomyocytes through the PI3K/AKT signaling pathway to achieve anti-cardiac H/R and exert myocardial protective effects.

### Activation of PI3K/AKT pathway by SPostC maintains mitochondrial dynamics homeostasis by regulating Drp1 and Opa1 through HIF-1α

**Fig. 4:**
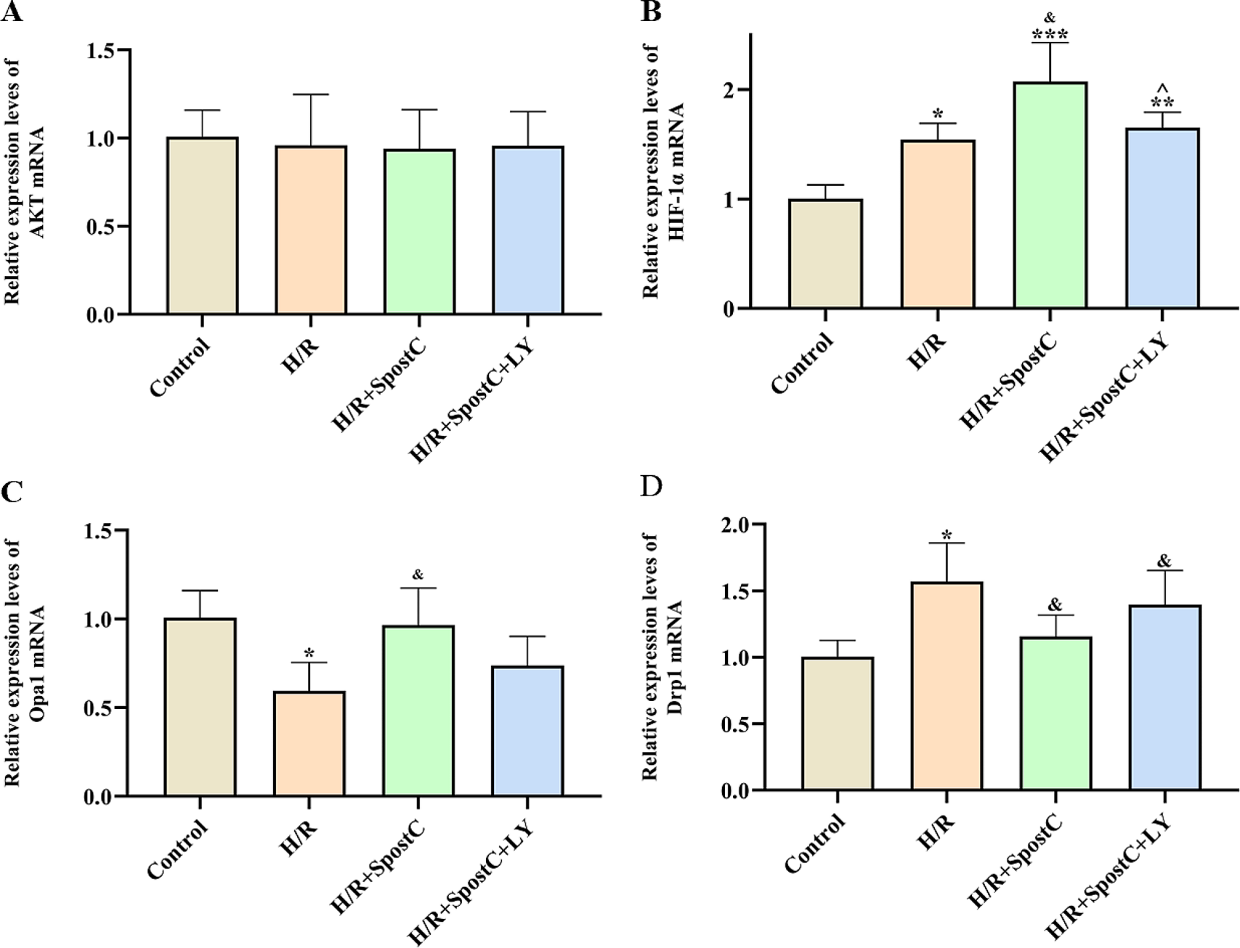
qRT-PCR was used to determine the mRNA expression of AKT, HIF-1α, Opa1 and Drp1. (**A**)Expression levels of AKT mRNA. (**B**) Expression levels of HIF-1α mRNA. (**C**) Expression levels of Opa1 mRNA. (**D**) Expression levels of Drp1 mRNA. *: Compared with Control group, *P* < 0.05; **: Compared with Control group, *P* < 0.01; ***: Compared with Control group, *P* < 0.001; &: Compared with H/R group, *P* < 0.05; ^: Compared with H/R + SpostC group, *P* < 0.05

The dynamic balance between fusion and fission is an important part of mitochondrial dynamics, and optic atrophy 1 (OPA1) and dynamin-related protein 1 (Drp1) are key regulators. The mRNA expression of AKT, HIF-1α Drp1and Opa1was measured by qRT-PCR (Fig. 4). We found that there was no difference in the expression of AKT mRNA among all groups. The mRNA expression of HIF-1α and Drp1 increased, and the mRNA expression of Opa1 decreased after H/R injury in cardiomyocytes. Compared with the H/R group, SpostC significantly increased the mRNA expression of HIF-1α and Opa1, and decreased the mRNA expression of Drp1. However, HIF-1α mRNA expression was lower in the H/R + SpostC + LY group than in the H/R + SpostC group, and there was no difference in Opa1 mRNA expression between the H/R group and the H/R + SpostC + LY group. The results suggest that SpostC could increase the transcription of HIF-1α to regulate transcription of mitochondrial fusion and fission-associated proteins, through the PI3K/AKT pathway.

### Activation of the PI3K/AKT pathway regulates the expression of Drp1 and Opa1 by increasing the expression of HIF-1α

**Fig. 5:**
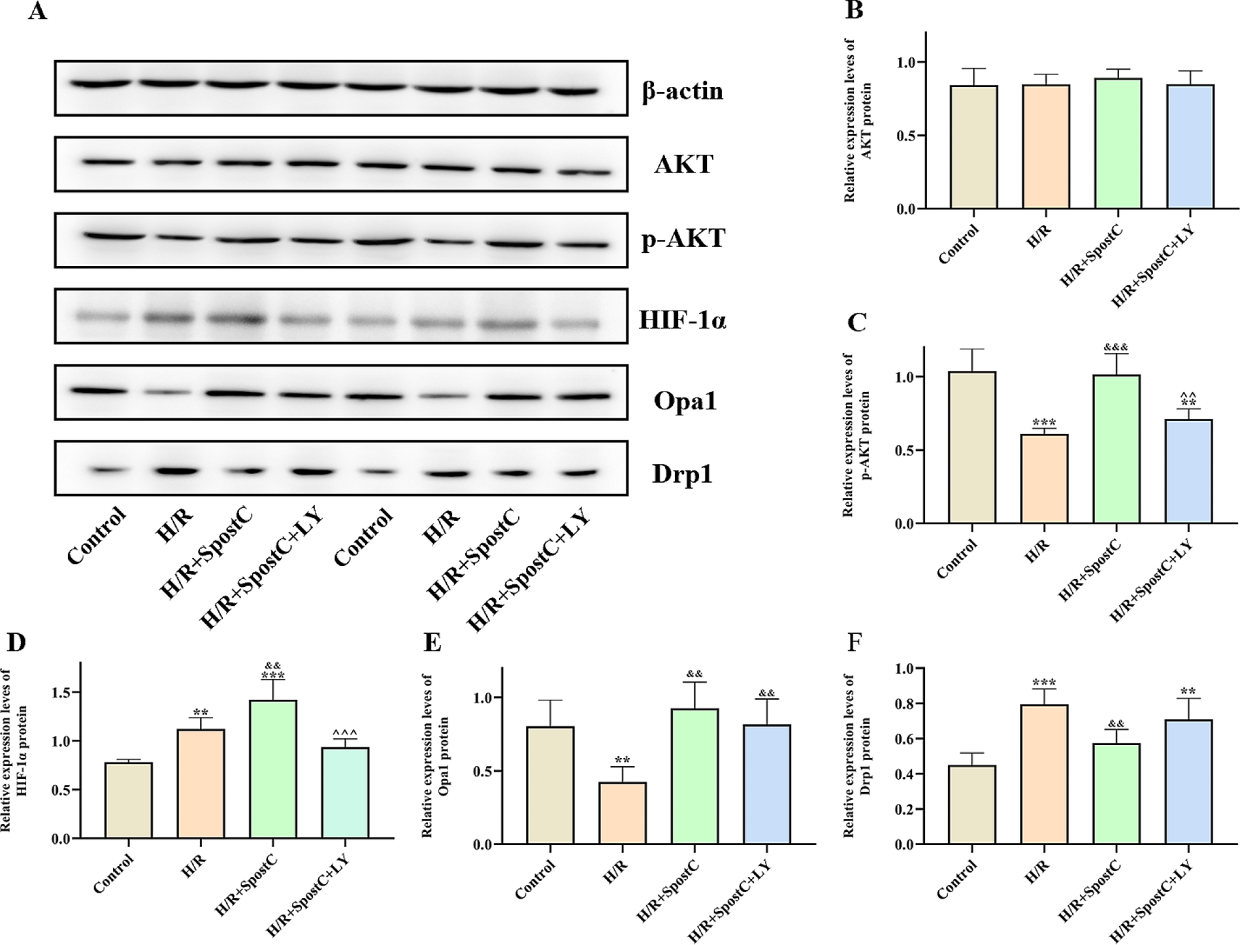
Protein expression levels of AKT, p-AKT, HIF-1α, Opa1 and Drp1 were detected by Western Blot. (**A**) Protein bands. (**B**) Expression of AKT. (**C**) Expression of p-AKT. (**D**) Expression of HIF-1α. (**E**) Expression of Opa1. (**F**) Expression of Drp1. **: Compared with Control group, *P* < 0.01; ***: Compared with Control group, *P* < 0.001; &&: Compared with H/R group, *P* < 0.01; &&&: Compared with H/R group, *P* < 0.001; ^^: Compared with H/R + SpostC group, *P* < 0.01; ^^^: Compared with H/R + SpostC group, *P* < 0.001

Drp1 and Opa1 are key proteins that regulate the homeostasis of mitochondrial dynamics. The protein expression levels of AKT, p-AKT, HIF-1a, Opa1 and Drp-1 in each group were measured by Western blot (Fig. 5). The protein expression levels of p-AKT, HIF-1α and Opa1 in the SpostC group were significantly higher than those in the H/R group, while the protein expression levels of Drp1 were lower. Protein expression levels of p-AKT and HIF-1α in the S + LY group were lower than those in the SpostC group, while AKT protein expression levels did not change significantly among the four groups. Our results show that SpostC activates the PI3K/AKT pathway and increases the phosphorylation level of AKT, which increases HIF-1α expression to regulate mitochondrial fission and fusion-related proteins.

## Discussion

**Fig. 6:**
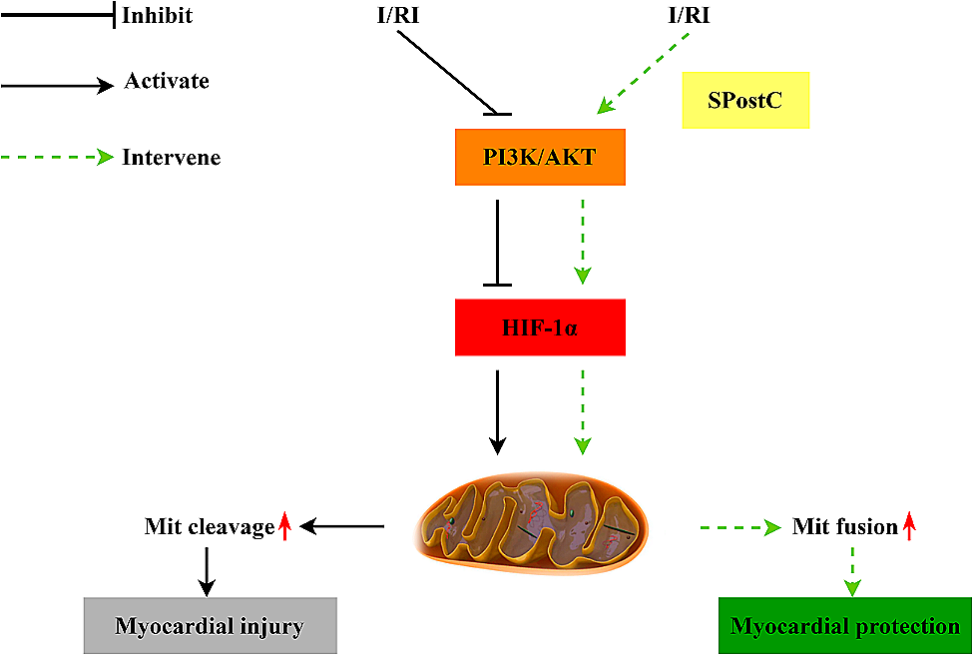
Schematic diagram of the research process

In this study, H9C2 cardiomyocytes were cultured and established as an H/R model, and SpostC and the PI3K/AKT blocker (LY294002) were administered to investigate whether SpostC promotes HIF-1α-regulated mitochondrial fission and fusion to maintain mitochondrial dynamic balance by activating the PI3K/AKT pathway. It was demonstrated that SpostC significantly improved cell survival, reduced apoptosis and significantly improved myocardial mitochondrial membrane potential after myocardial H/R injury, but this cardioprotective effect was attenuated by blockers of PI3K/AKT. The experimental results showed that SPostC counteracts myocardial H/R injury by activating the PI3K/AKT pathway and increasing the phosphorylation level of AKT, which mediates HIF-1α regulation of mitochondrial fusion and fission-related proteins (Opa1/Drp1)(Fig. 6).

Sevoflurane is considered by most scientists to be a landmark inhalational anesthetic widely used in various clinical procedures. The protective effect of sevoflurane on ischemic myocardium has been found to be closely related to its mode of administration, such as sevoflurane pretreatment (SPC) or sevoflurane post-treatment (SpostC) [19–21]. This study confirms that the cardioprotective effect of SpostC is achieved by activating the PI3K/AKT pathway and increasing the phosphorylation level of AKT, which mediates downstream HIF-1α.

Mitochondria are dynamic organelles that change their morphology through constant fission and fusion [22], and through this dynamic change, mitochondrial homeostasis is achieved and its metabolic function is maintained [23]. Researches have confirmed that dynamin-related protein 1 (Drp1) and optic atrophy protein 1 (Opa1) play important roles in mitochondrial fission and fusion [24, 25]. Our previous studies have demonstrated that SPostC maintains mitochondrial homeostasis through HIF-1α regulation of Drp1 and OPA1 to mitigate I/RI [11]. The results of the present study also showed again that SPostC increased Opa1 and decreased Drp1 expression levels via HIF-1α to maintain mitochondrial kinetic homeostasis and counteract cardiomyocyte H/R injury.

The imbalance of myocardial energy metabolism is the initial link in the pathogenesis of I/RI. The characteristics of myocardial energy metabolism during I/RI are as follows: Early in myocardial ischemia, energy metabolism changes from aerobic oxidation in the mitochondria to glycolysis, and adenosine triphosphate (ATP) produced by glycolysis becomes the only source of energy to keep the myocardial cells alive. After reperfusion, the inhibition of the glycolytic process is diminished as lactate and inorganic phosphate are washed out, while damage to the mitochondria from ischemia results in the inability of the mitochondria to use oxygen efficiently for aerobic oxidation during reperfusion. As a result, cardiac myocytes continue to rely on glycolysis for energy for a considerable period of time after reperfusion [26]. It has been shown that increasing circulating glycolytic substrate concentrations during myocardial I/RI attenuates myocardial cell injury and promotes recovery of myocardial function after reperfusion [27]. This suggests that glycolysis during I/RI is an important source of energy required by the myocardium [28].

HIF-1α, a transcription factor, is a central component of the oxygen-sensitive machinery of mammalian cells and has been shown to be an important regulator of the hypoxic and ischemic response [29]. Under normoxia, HIF-1α is degraded by proline hydroxylase; when hypoxia occurs, HIF-1α accumulates in large quantities due to inhibition of degradation. HIF-1α translocates to the nucleus and forms a dimeric complex with HIF-1β, which binds to hypoxia-sensitive genes and regulates downstream target genes [30]. HIF-1α is involved in the regulation of key enzymes of glycolysis and plays an important role in metabolic processes [31, 32]. Therefore, HIF-1α may play a cardioprotective role in the anti-I/RI process through several aspects including regulation of energy metabolism and maintenance of mitochondrial dynamic balance.

The PI3K/AKT pathway is involved in a variety of biological processes including the cell cycle, apoptosis, angiogenesis and glucose metabolism [13]. AKT is thought to be the central mediator of the PI3K/AKT signal pathway and is involved in the phosphorylation of many important downstream targets [33]. Previous studies have shown that HIF-1α is regulated by the PI3K/AKT/mTOR [16] and PI3K/AKT/FRAP signal pathway [34]. We found that the levels of p-AKT, HIF-1α and opa1 were significantly increased after SpostC, while the levels of p-AKT, HIF-1α and opa1 were significantly decreased after using PI3K inhibitor. It is suggested that SpostC not only increases HIF-1α mRNA and protein expression through activation of PI3K/AKT, but may also increase the stability of HIF-1α, which is consistent with previous findings [34]. In turn, it promoted mitochondrial fusion and reduced mitochondrial fission, therefor exerting a cardioprotective effect by maintaining mitochondrial kinetic homeostasis.

Our team’s previous experiments have confirmed the myocardial protective effect of SPostC in practical clinical applications [35]. We further studied the mechanism of effect of SPostC and found that SPostC regulates Drp1 and OPA1 through HIF-1α to maintain mitochondrial homeostasis and resistance I/RI [10]. This study is to explore the signal regulation mechanism of SPostC myocardial protective effect.

There are several limitations to this study. We used H9C2 cardiomyocytes rather than primary cardiomyocytes in culture and further validation at the animal level is required. The PI3K blocker LY294002 was chosen to validate the role of the PI3K/AKT pathway, rather than blocking PI3K and AKT separately. In the next step, we will carry out single-cell sequencing and animal experiments to further study the fruit in order to solve the shortcomings of this study.

## Conclusions

In conclusion, this study confirmed that SpostC mediates HIF-1α-regulated mitochondrial fission and fusion-related protein expression to maintain mitochondrial dynamic balance by activating the PI3K/AKT pathway and increasing AKT phosphorylation, thereby attenuating myocardial I/R injury.

### Electronic supplementary material

Below is the link to the electronic supplementary material.

Supplementary Material 1

## Data Availability

All data generated or analyzed during this study are included in this published article.
