# Supplementary figures and images for: Sevoflurane postconditioning attenuates cardiomyocytes hypoxia/reoxygenation injury via PI3K/AKT pathway mediated HIF-1α to regulate the mitochondrial dynamic balance
Source: BMC Cardiovasc Disord. 2024 May 29;24:280. doi: 10.1186/s12872-024-03868-1 (PMC11134705; doi:10.1186/s12872-024-03868-1)

$\beta$ -actin

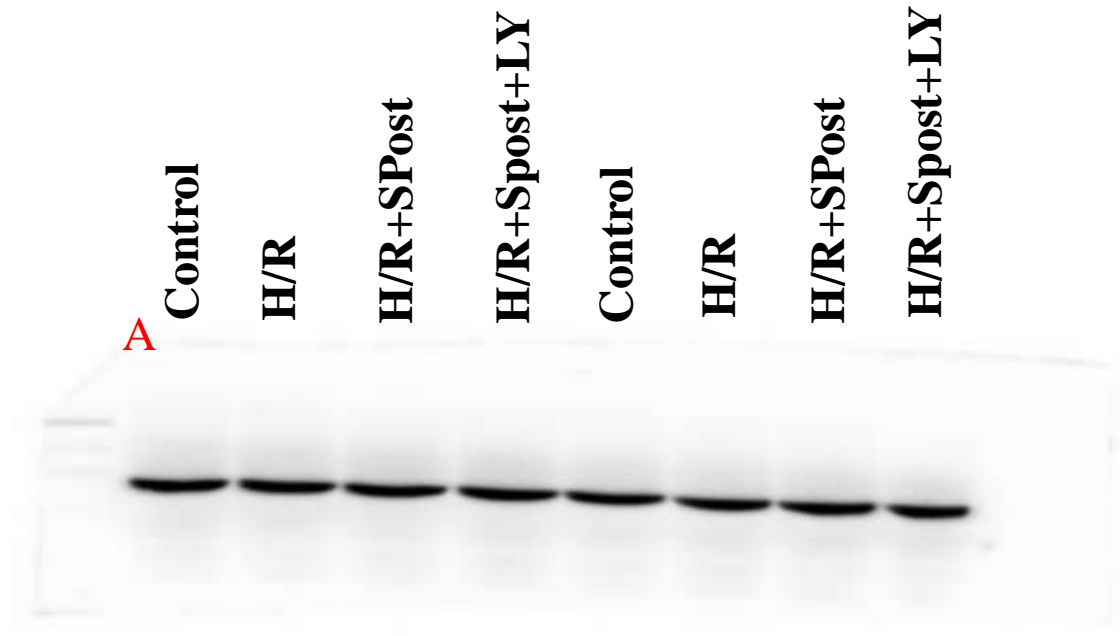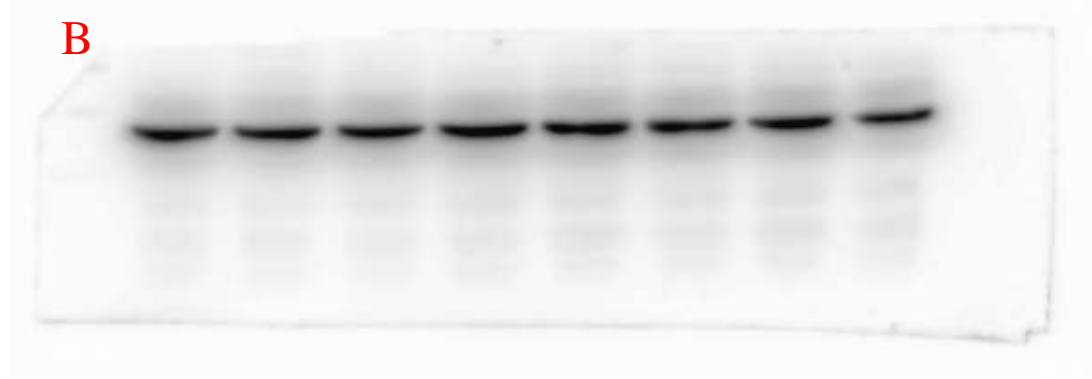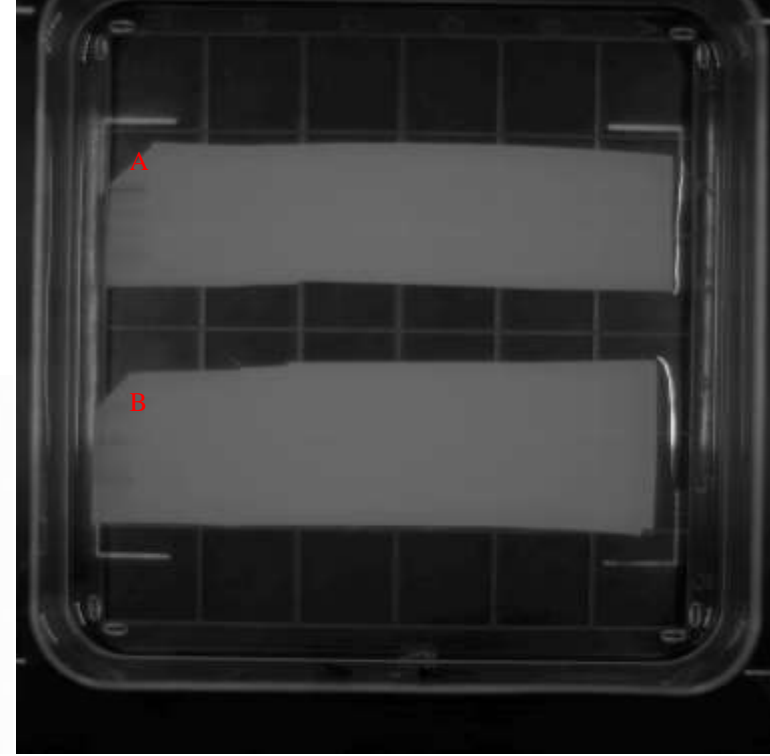

Akt1

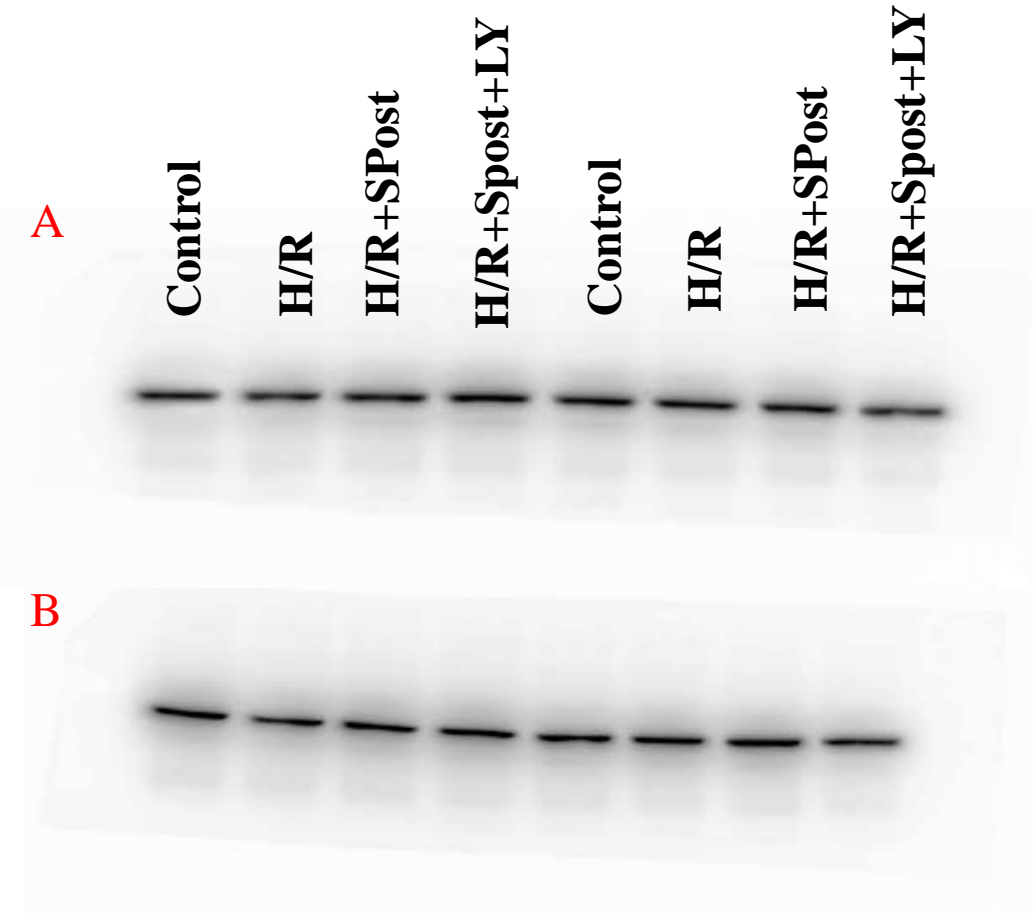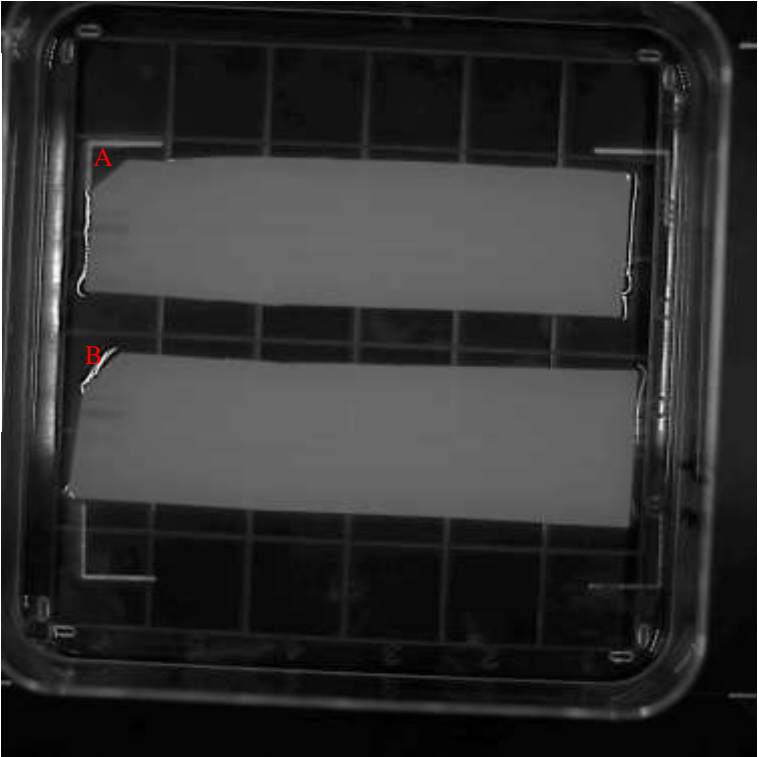

P-Akt

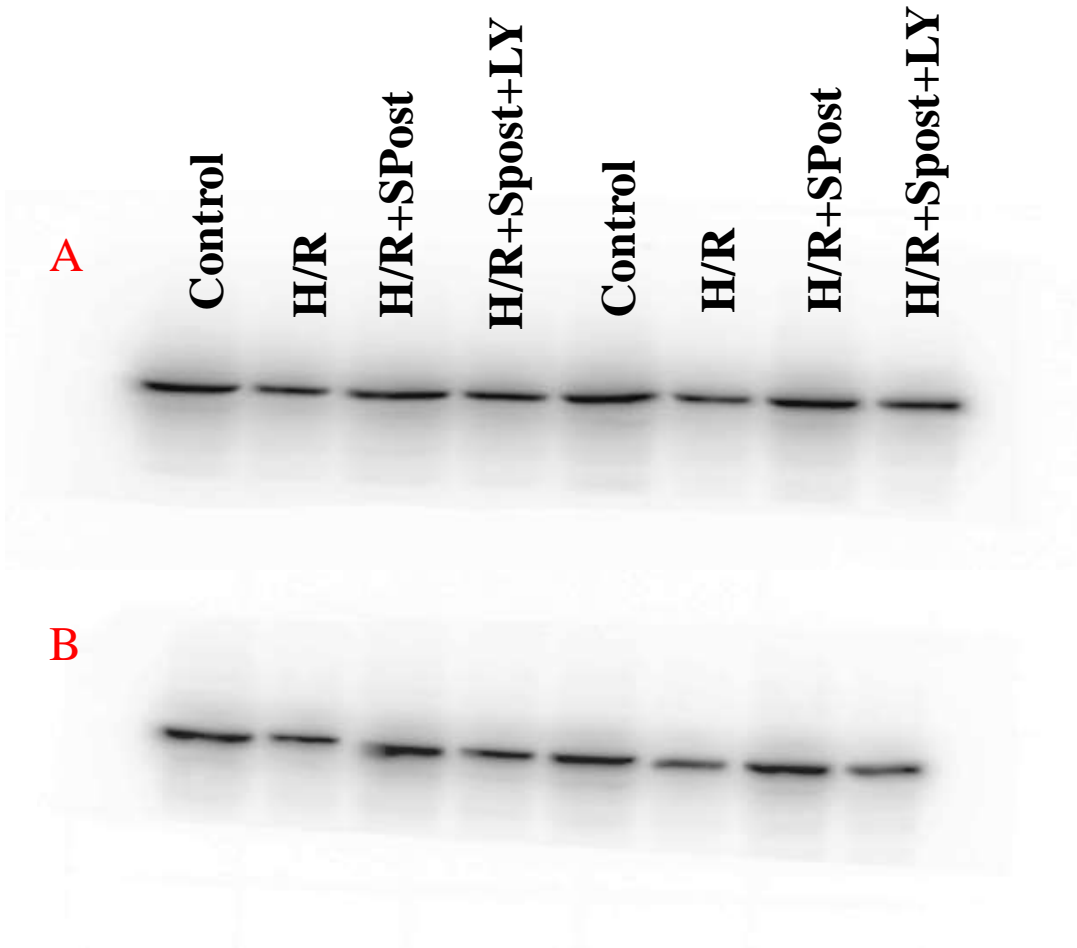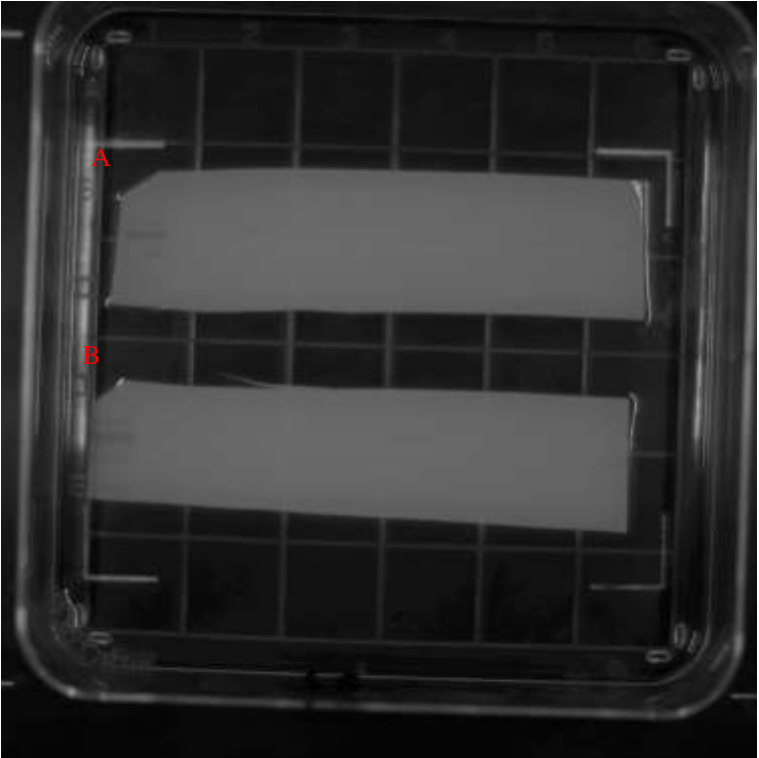

**Hif-1 $\alpha$**

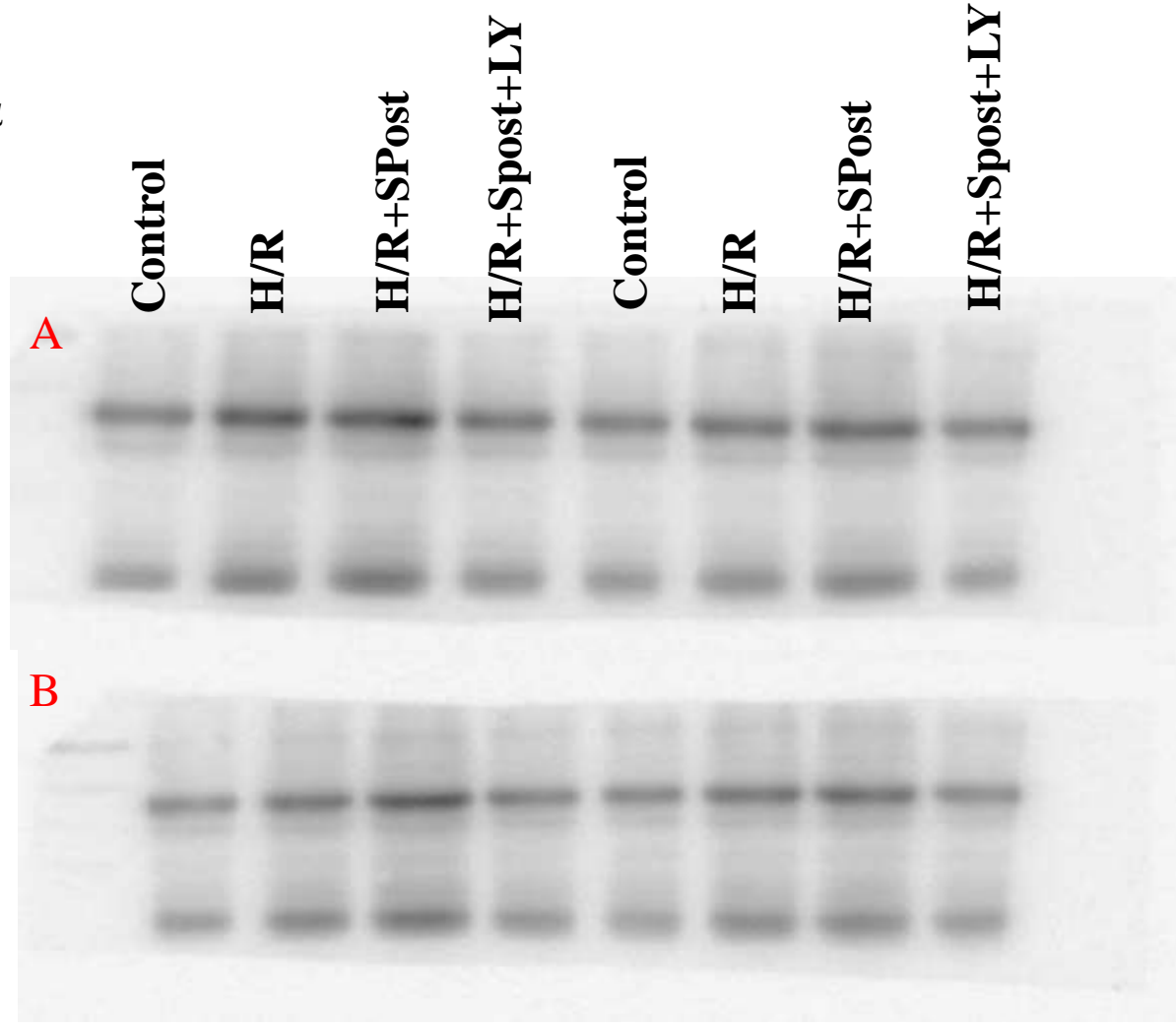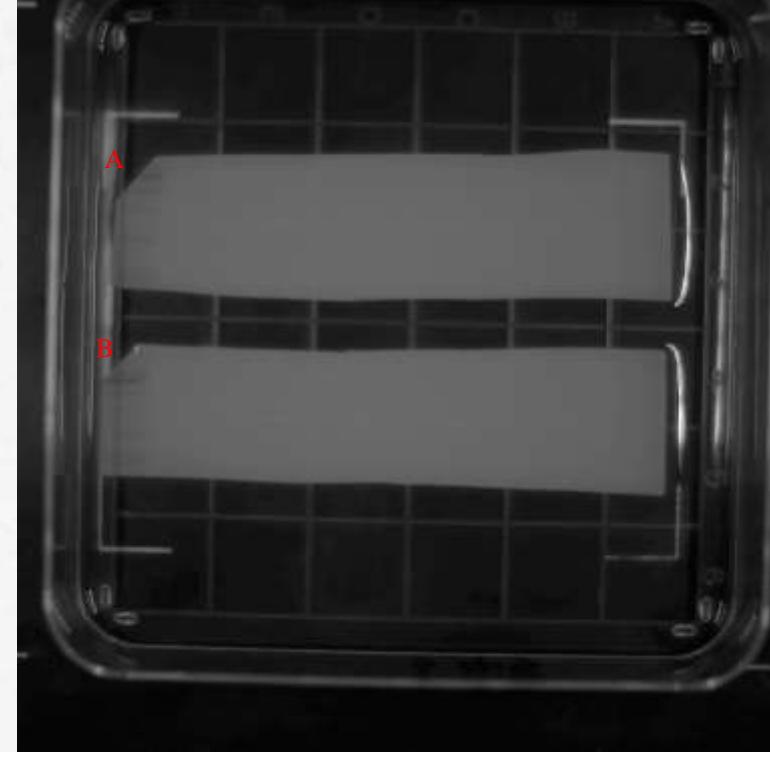

OPA1

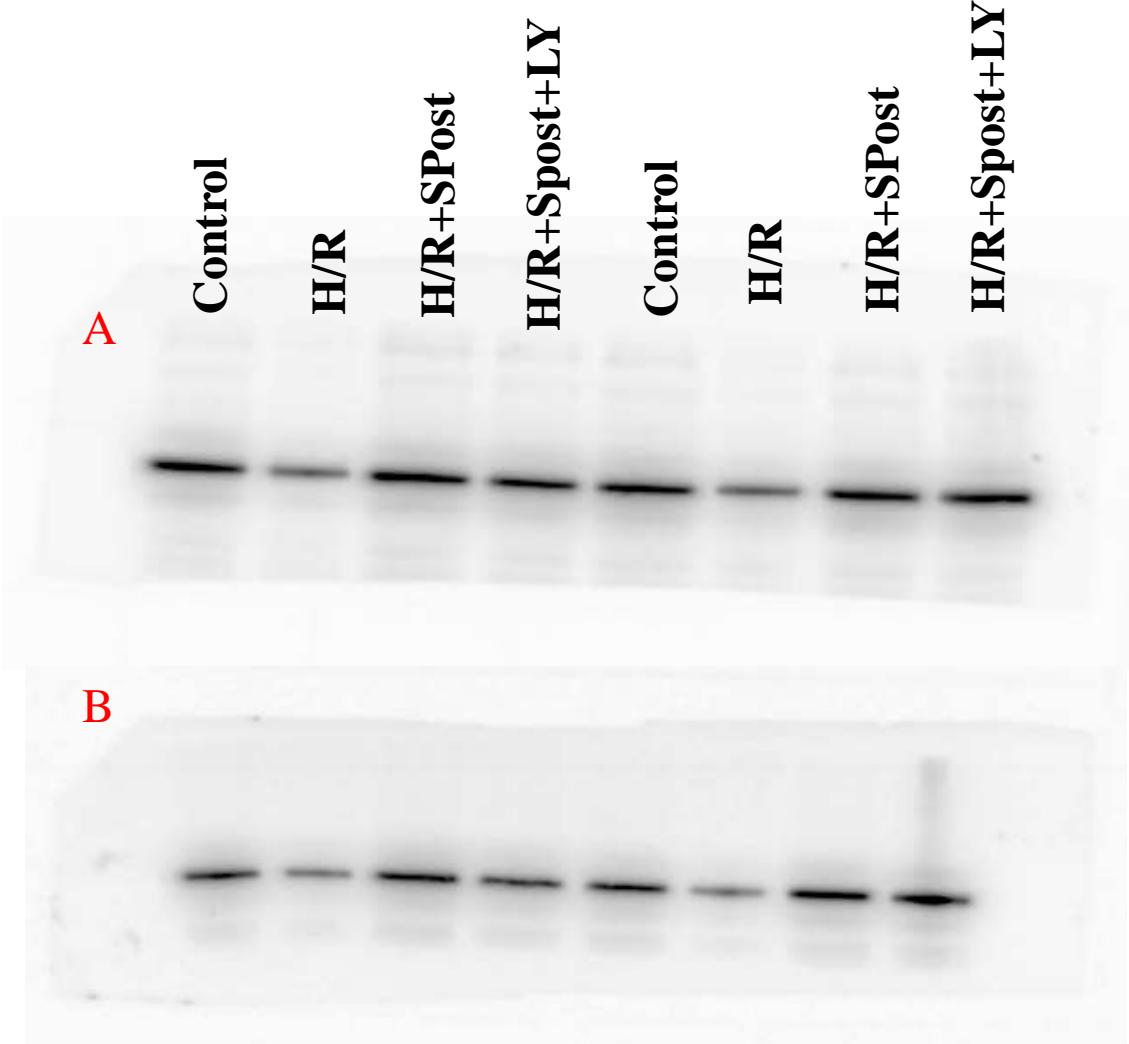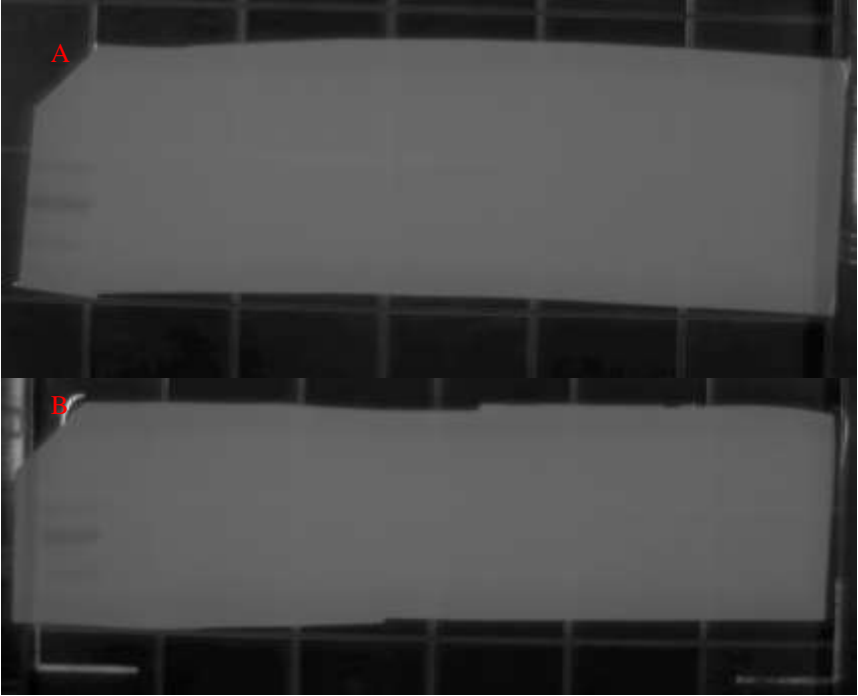

Drp1

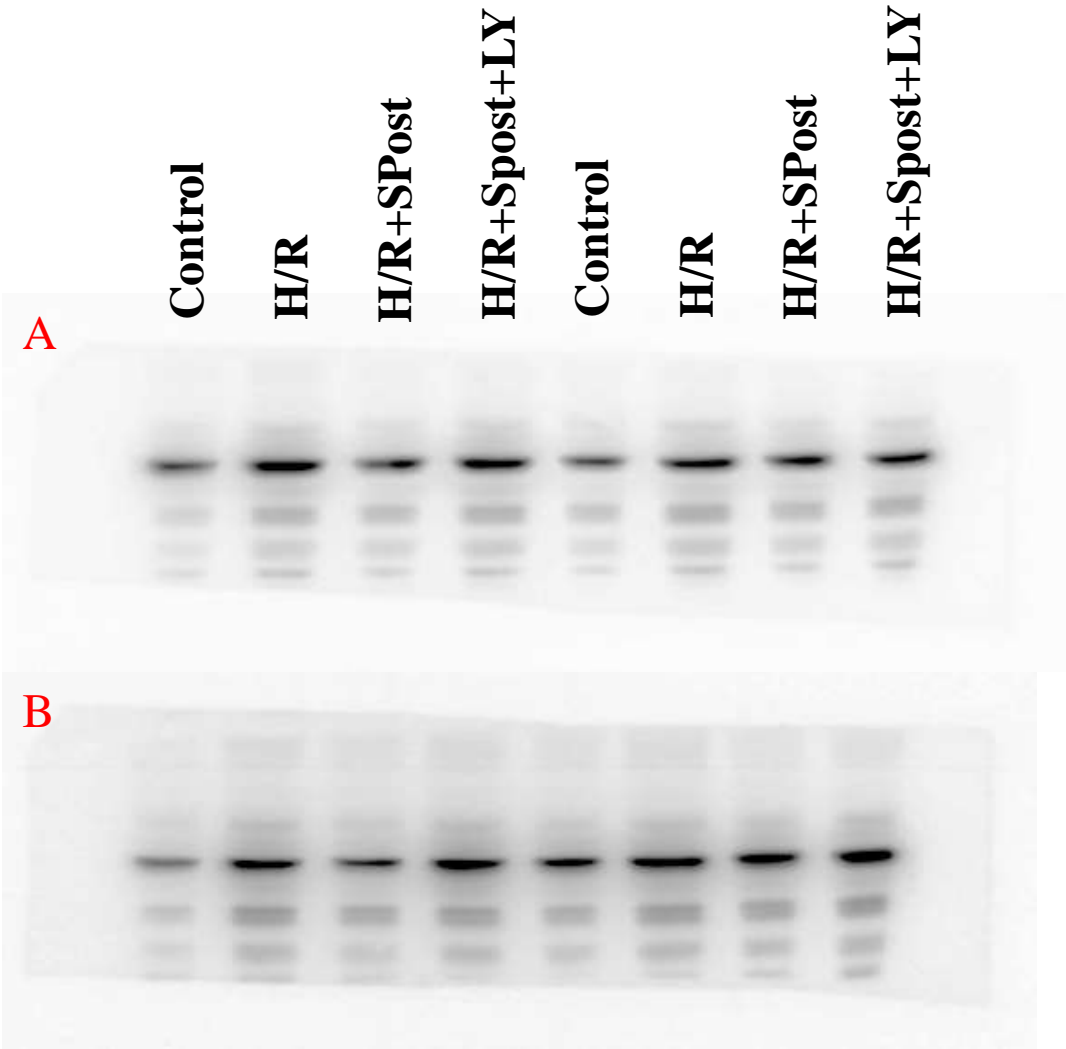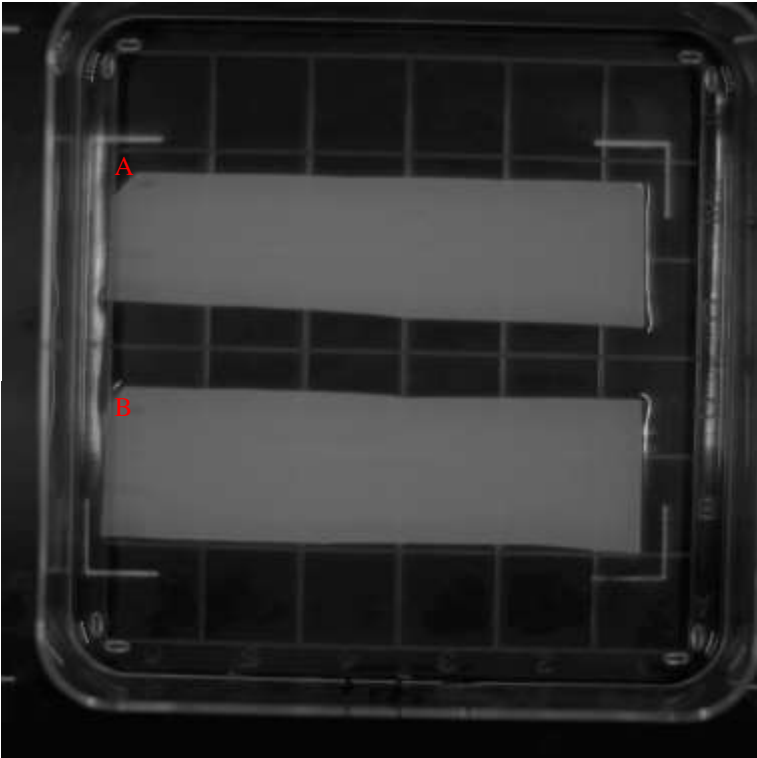

Supplement: Supplementary file 1 — Supplementary Material 1 [file 12872_2024_3868_MOESM1_ESM.pdf]
